# Chm-1 gene-modified bone marrow mesenchymal stem cells maintain the chondrogenic phenotype of tissue-engineered cartilage

**DOI:** 10.1186/s13287-016-0328-x

**Published:** 2016-05-05

**Authors:** Zhuoyue Chen, Jing Wei, Jun Zhu, Wei Liu, Jihong Cui, Hongmin Li, Fulin Chen

**Affiliations:** Laboratory of Tissue Engineering, Faculty of Life Science, Northwest University, 229 TaiBai North Road, Xi’an, Shaanxi Province 710069 P.R. China; Provincial Key Laboratory of Biotechnology of Shaanxi, Northwest University, 229 TaiBai North Road, Xi’an, Shaanxi Province 710069 P.R. China

**Keywords:** Chondromodulin-1, Mesenchymal stem cells, Chondrocytes, Cartilage tissue

## Abstract

**Background:**

Marrow mesenchymal stem cells (MSCs) can differentiate into specific phenotypes, including chondrocytes, and have been widely used for cartilage tissue engineering. However, cartilage grafts from MSCs exhibit phenotypic alternations after implantation, including matrix calcification and vascular ingrowth.

**Methods:**

We compared chondromodulin-1 (Chm-1) expression between chondrocytes and MSCs. We found that chondrocytes expressed a high level of Chm-1. We then adenovirally transduced MSCs with *Chm-1* and applied modified cells to engineer cartilage in vivo.

**Results:**

A gross inspection and histological observation indicated that the chondrogenic phenotype of the tissue-engineered cartilage graft was well maintained, and the stable expression of Chm-1 was detected by immunohistological staining in the cartilage graft derived from the Chm-1 gene-modified MSCs.

**Conclusions:**

Our findings defined an essential role for Chm-1 in maintaining chondrogenic phenotype and demonstrated that Chm-1 gene-modified MSCs may be used in cartilage tissue engineering.

## Background

Cartilage regeneration and repair is often needed in orthopedic or plastic and reconstructive surgery for the treatment of cartilaginous defects and malformations. Unlike other self-repairing tissues, cartilage is an avascular tissue characterized by a low cell density and limited nutrient supply [1, 2]. Because of the limited regenerative capacity of cartilage, the treatment of various cartilaginous lesions remains a challenge to clinicians.

Tissue engineering provides an optimized alternative for cartilage regeneration and repair by combining chondrogenic cells and a scaffold [3, 4]. Chondrocytes are most commonly used for cartilage tissue engineering. However, the harvesting of a cartilage biopsy to obtain primary chondrocytes may cause donor site morbidity, and chondrocytes will dedifferentiate and lose their chondrogenic phenotype during monolayer expansion [5]. Compared with chondrocytes, marrow mesenchymal stem cells (MSCs) could be easily isolated, expanded, and directed to differentiate into mesodermal lineages, including bone, cartilage, and adipose tissue [6, 7]. MSCs may undergo chondrogenic differentiation with the induction of transforming growth factor beta (TGF-β), especially under micropellet culture condition [8], and great efforts have been made to use MSCs for cartilage tissue engineering [9–11]. However, cartilage grafts from MSCs undergo gradual histological changes after implantation, including chondrocyte hypertrophy, extracellular matrix calcification, and vascular invasion, which may significantly influence the treatment outcome of cartilage defects [12, 13].

Coculturing with chondrocytes was usually employed to maintain the stable chondrogenic phenotype of MSCs. The approach could improve collagen type II and glycosaminoglycan expression as well as the deposition of MSCs. The mechanism was attributed to signaling via direct cell–cell contacts [14–16] and paracrine factors secreted by chondrocytes [17, 18]. Kang et al. [19] demonstrated that a 1:1 ratio of chondrocytes to MSCs can be used to engineer phenotypically stable cartilage, and obvious vascular invasion could not be observed 6 weeks after in-vivo implantation. However, engineering cartilage with co-seeding of chondrocytes and MSCs still requires the surgical harvesting of cartilage biopsy to obtain chondrocytes [20].

Chondromodulin-1 (Chm-1) is a glycoprotein with 25 kDa molecular weight and is found highly expressed in cartilage tissue [21]. Chm-1 could inhibit the endothelial cell proliferation and tube morphogenesis, induce apoptisis of vascular endothelial cells in vitro, as well as inhibit angiogenesis in the chick chorioallantoic membrane [22–24]. Mature cartilage contains considerable amounts of Chm-1, is avascular, and its extracellular matrix does not calcify. Meanwhile healing cartilage from MSCs within the cartilage lesions of the knee joint lacked Chm-1 expression, and exhibited excessive ossification and vascularization [25]. Based on these findings, we may deduce that the phenotypic drift of cartilage grafts from MSCs after in-vivo implantation is due to the low expression of Chm-1 in MSCs.

In the current experiment, we first compared Chm-1 expression profiles in MSCs and chondrocytes. We then engineered phenotypically stable cartilage grafts from Chm-1 gene-modified MSCs. Coral has an interconnective porous structure and good osteoconductive activity which are suitable for blood vessel invasion and tissue ossification [26–28]. We chose coral as the cell-seeding scaffold to investigate the critical effect of Chm-1 on antivascularization and maintaining the chondrogenic phenotype of tissue-engineered cartilage with MSCs.

## Methods

All reagents were purchased from Sigma-Aldrich (St. Louis, MO, USA) unless otherwise specified.

### Isolation and culture of MSCs and chondrocytes

Rabbit MSCs were isolated and cultured as reported previously [28]. New Zealand rabbits (1 month old) were obtained from the animal holding unit of Four Military Medical University (FMMU, Xi'an, Shaanxi Province, P.R. China) and samples of bone marrow were harvested in accordance with IACUC approval from Northwest University, Xi’an, P.R. China. Briefly, the obtained marrow was suspended and cultured in Dulbecco’s modified Eagle medium (DMEM; Gibco BRL, Grand Island, NY, USA) containing 10 % fetal bovine serum (FBS), 272 μg/ml l-glutamine, and 100 U/ml penicillin/streptomycin. The media were changed every 3 days. Before the cells formed a confluent monolayer, they were digested using trypsin 0.25 % and harvested by centrifugation, and cells of passage 2 were used for the experiment. The cell density was adjusted to 5 × 10^7^ cells/ml with medium before cell seeding.

Rabbit chondrocytes were isolated and cultured according to the method described by Wu et al*.* [29]. All New Zealand rabbits were anesthetized with ketamine (40 mg/kg, intramuscularly) and xylazine (5 mg/kg, intramuscularly). After aseptic preparation, the auricle cartilage from ear roots was dissected and cut into pieces of approximately 2 mm^3^ after being rinsed three times with phosphate-buffered saline (PBS) supplemented with 100 U/ml penicillin and 100 U/ml streptomycin; the cartilage samples were digested with 0.2 % collagenase type II (Gibco) in DMEM (Gibco) at 37 °C for 12 hours. The digested cell suspension was filtered through a 250 mm nylon mesh filter to remove matrix debris and was centrifuged at 1000 rpm for 5 minutes; the resulting cell pellet was washed twice with PBS and resuspended with DMEM containing 10 % FBS, l-glutamine (272 μg/ml), and ascorbic acid (5 μg/ml). The medium was changed every 3 days. The chondrocytes were subcultured twice, collected by trypsin digestion, and suspended in culture medium at a density of 5 × 10^7^ cells/ml for seeding.

### RNA isolation and reverse transcription-PCR

The expression levels of *Chm-1*, *Col II*, and *AGG* in chondrocytes and MSCs were compared by reverse transcription-PCR (RT-PCR). Total RNA was isolated from chondrocytes and MSCs using TRIzol Reagent (Invitrogen, Carlsbad, CA, USA). For cDNA synthesis, RT-PCR was performed using the Takara RT-PCR Kit (Takara, Dalian, China). The reaction product cDNA was used as a template for PCR amplification. The PCR conditions were as follows: initial denaturation at 94 °C for 3 minutes; 30 cycles at 94 °C for 40 seconds, 60 °C for 40 seconds, and 72 °C for 80 seconds; and a final extension at 72 °C for 5 minutes. The PCR products were visualized on 1.5 % agarose gels. *GAPDH* was used as an internal control. The primer sequences used for this analysis are presented in Table 1. Band intensity was quantified using Bandscan software. The gray values of bands were normalized relative to those of *GAPDH*. The gray values were expressed in relation to the control and presented as means ± SD from four independent experiments.

**Table 1:** Primer sequences for reverse transcription-PCR

Gene	Primers	Product size (base pairs)
*GAPDH*	TCACCATCTTCCAGGAGCGA	293
*AGG*	CACAATGCCGAAGTGGTCGT GGTCGTGGTGAAAGGTGTTGT	315
*Col II*	GCAGACGCATGAAGGCAAGTT AGCAGCAGCACGTGTGGTT	97
*Chm-1*	ATCTGGACGTTGGCAGTGTTG CCGCTCGAGCATGACCGAGAACTCGGACA	1022
	CCGGAATTCGCACCTGATACGCAAAGTGA	

### Western blot analysis

Western blot analysis was carried out for Chm-1, Col II, and AGG expression of MSCs and chondrocytes. Equal amounts of protein extracts (30 μg/lane) were separated by SDS-PAGE and transferred to the nitrocellulose membrane. Nonspecific binding was blocked with TBS buffer (50 mM Tris/HCl and 150 mM NaCl) containing 5 % (w/v) skimmed milk for 2 hours at room temperature. The membranes were then incubated with primary antibodies (1:1000 (v/v) for GAPDH, 1:1000 (v/v) for Chm-1, 1:1000 (v/v) for Col II, and 1:1000 (v/v) for AGG; Santa Cruz, Santa Cruz, California, USA) for 2 hours at 37 °C. After washing with TBS containing 0.05 % Tween-20 (TBST) three times, the membranes were incubated for 1 hour at 37 °C with secondary antibodies conjugated with horseradish peroxidase diluted 1:1000 in TBST. Finally, the membranes were treated with enhanced chemoluminescence (ECL) reagent (Santa Cruz) and exposed to Kodak X-ray film. GAPDH acted as internal control.

### MSCs modified with *Chm-1* gene

*Chm-1* cDNAs were amplified from rabbit chondrocytes by PCR using the primers presented in Table 1. The PCR products were subcloned into the pDC316 expression adenovirus vector (pDC316-Chm-1) after restriction enzyme digestion. According to the manufacturer’s instructions for the AdMax Kit D (Microbix Biosystems Inc.,Toronto, ON, Canada), HEK 293 producer cells were cotransfected with pBHGlox E1, 3Cre, and expression adenovirus vector (pDC316-Chm-1) to obtain the adenovirus-containing Chm-1 gene (Ad5-Chm-1).

Second-passage rabbit MSCs were transduced with an adenovirus containing either green fluorescent protein (GFP) (Ad5-GFP) or Chm-1 (Ad5-Chm-1) for 72 hours at a multiplicity of infection (MOI) of 1000 plaque-forming units (PFU)/cell. The efficiency of adenovirus gene transfer in MSCs was evaluated under a fluorescence microscope 72 hours after transfection.

### Expression of Chm-1 in Ad5-Chm-1-transfected MSCs

Total RNA was isolated from MSCs before infection and 72 hours after infection using TRIzol Reagent (Invitrogen). For cDNA synthesis, the total RNA was reverse-transcribed using the Takara RT-PCR Kit for RT-PCR. The reaction product cDNA was used as a template for PCR amplification. The PCR conditions were as follows: initial denaturation at 94 °C for 3 minutes; 30 cycles at 94 °C for 40 seconds, 60 °C for 40 seconds, and 72 °C for 80 seconds; and a final extension at 72 °C for 5 minutes. PCR products were visualized on 1.5 % agarose gels. *GAPDH* was used as the internal control. The primer sequences used for this analysis are presented in Table 1. The gray values of bands were normalized relative to those of *GAPDH*. The gray values were expressed in relation to the control and presented as means ± SD from four independent experiments. To confirm the bioactivity of transgenic *Chm-1*, the expression levels of Chm-1, Col II, and AGG in Ad5-Chm-1-transfected MSCs (T-MSCs) were measured by western blot analysis. The western blot analysis was carried out as described previously. Furthermore, the Chm-1 and Col II proteins were detected by immunofluorescence. Briefly, the MSCs, chondrocytes, and T-MSCs were washed three times with PBS (pH 7.4). Cells were fixed for 10 minutes by incubating in 4 % formaldehyde in PBS followed by further washing and preincubation with 1 % bovine serum albumin (BSA) for 30 minutes. Incubation was with anti-Chm-1 antibody (Santa Cruz) and anti-Col II antibody (Santa Cruz) for 20 minutes at room temperature. Next, the samples were rinsed in PBS followed by incubation with Cy3-conjugated antimouse secondary antibody (Calbiochem, Darmstadt, Germany) for 20 minutes at room temperature, PBS washing, and finally staining with 5 mg/ml Hoechst 33342 for 30 minutes. The fluorescence images from stained samples were obtained using a confocal laser scanning microscope (FV1000; Olympus Corporation, Tokyo, Japan).

### Construction of cell–scaffold complex

Natural coral (Gonophoresduofaciata, Hainan, China) was carefully molded into the shape of a tube that was 8 mm in diameter and 2 mm thick. The material was treated as described previously [30]. Briefly, it was immersed in 50 mg/ml sodium hypochlorite for 14 days, and the medium was changed every other day to remove foreign protein in the coral. The scaffold was then washed with distilled water and autoclaved before use.

In preparation for cell seeding, empty scaffolds were prewetted in culture medium for 10 minutes. Cells were then pipetted onto each scaffold to achieve a final seeding number of 2 × 10^6^ cells in 40 μl suspensions. Each scaffold was seeded with cells which were i) chondrocytes, ii) 1:1 mixture of MSCs and chondrocytes, iii) MSCs, and iv) T-MSCs. The cell–scaffold complexes were placed into dishes and moved to the incubator for 4 hours to ensure that most cells adhered to the scaffolds. Then, 2 ml of medium was carefully added around the complexes. Twelve hours later, an additional 10 ml of medium was added. The composites were then incubated for 5 days to allow for cell attachment. Prior to implantation, the scaffolds were rinsed in sterile PBS, stained with Hoechst 33342, and observed through a fluorescence microscope (Nikon, Tokyo, Japan). Twelve hours and 5 days after cell seeding, a PicoGreen DNA quantitation assay [31] was used to monitor cell-seeding efficacy and proliferation on coral scaffolds (*n* = 4). The DNA quantitation of  2 × 10^6^ cells before seeding acted as controls.

### Subcutaneous implantation in nude mice

Eight BALB/c nude mice (6 weeks old, from the animal holding unit of FMMU) were used for the experiment. The animals were acclimated for 1 week before the surgery and monitored for general appearance, activity, excretion, and weight. All procedures were approved by the IACUC of Northwest University. Before implantation, the nude mice were anesthetized by intraperitoneal injection of 10 % chloral hydrate (300 mg/kg). After aseptic preparation, the skin on the back was incised and a subcutaneous pocket was made. The coral–implant composite scaffolds loaded with cells (four kinds of composites: scaffold loaded with i) chondrocytes, ii) 1:1 chondrocytes and MSCs, iii) MSCs, and iv) T-MSCs) were implanted into eight animals (each animal received four kinds of implant). After 1 and 2 months of implantation, the animals (*n* = 4, at each time point) were sacrificed by neck dislocation and specimens were harvested. The specimens were observed by gross inspection and then fixed with 10 % phosphate-buffered formalin.

### Analyses of Chm-1 distribution and vascularization in newly formed tissue

After the specimens were fixed in 10 % phosphate-buffered formalin for 24 hours and demineralized in 5 % formic acid for 5 days, they were dehydrated in graded alcohols and embedded in paraffin before preparing sections 7 μm thick. The sections were stained with hematoxylin and eosin (H & E) and toluidine blue (TB) to evaluate the cartilaginous matrix. Finally, Masson’s trichrome staining (MTS) was utilized to detect the vascular structure [32].

Immunofluorescence was performed using the following primary antibodies: anti-Col II antibody (Santa Cruz) and anti-Chm-1 antibody (Santa Cruz). All incubations were performed in a humidified chamber. Next, the sections were rinsed in PBS followed by incubation with FITC-conjugated antimouse secondary antibody (Calbiochem). Finally, the sections were examined using a fluorescence microscope.

The blood vessel density was quantitatively analyzed from MTS of representative sections from each specimen. Vessels were identified by their luminal structure and the presence of red blood cells stained yellow within their boundaries. Vessels were counted from four random fields of each section under 200× magnification as the vessel number in each specimen. The vessel density in each group was determined by the average number of blood vessels from specimens (*n* = 4).

### Statistical analysis

Results are reported as the mean ± SD, and significance was determined using a probability value of *P* < 0.05. The significance of differences between groups was assessed using a two-way analysis of variance (ANOVA) with Tukey’s post-hoc analysis.

## Results

### Different expression of *Chm-1* gene in chondrocytes and MSCs

**Fig. 1:**
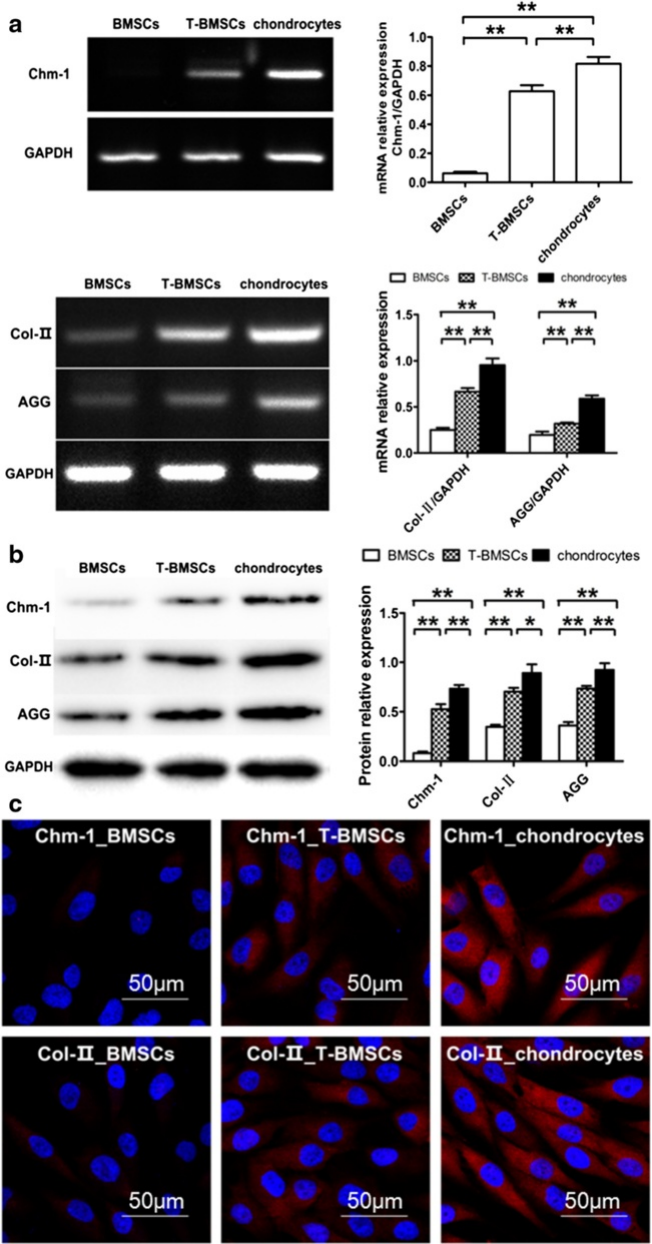
**a** RT-PCR evaluation of *Chm-1*, *Col II*, and *AGG* gene expression in chondrocytes, MSCs, and T-MSCs cultured in vitro. *GAPDH* was used as an internal control. Intensity levels showing *Chm-1*, *Col II*, and *AGG* mRNA expression levels in T-MSCs and chondrocytes were significantly higher than those of MSCs (**P < 0.01, mean ± SD, *n* = 4). **b** Western blot assay for Chm-1, Col II, and AGG expression. Chm-1, Col II, and AGG expression were significantly upregulated in T-MSCs and chondrocytes. Analysis of band intensities indicated that Chm-1, Col II, and AGG expression levels higher in T-MSCs and chondrocytes than the levels in MSCs (**P < 0.01, mean ± SD, *n* = 4). **c** Immunofluorescence examination showed cell transduction efficiency 5 days after transduction. Chm-1 and Col II protein expression was evaluated by immunofluorescence examination. Data are represented as mean ± standard deviation from four independent sets of experiments. *P ≤ 0.05, **P ≤ 0.01, and ***P ≤ 0.001  *BMSC* bone marrow mesenchymal stem cell, *Chm-1* Chondromodulin-1, *T-BMSC* Ad5-Chm-1 transfected BMSC

According to the RT-PCR results, both chondrocytes and MSCs expressed genes encoding cartilage-specific matrix proteins, including *Col II* (97 base pairs (bp)) and *AGG* (315 bp) (Fig. 1a). Importantly, high *Chm-1* gene (1000 bp) expression was found in chondrocytes, whereas expression of *Chm-1* gene was low in MSCs (Fig. 1a). Furthermore, Chm-1, Col II, and AGG protein levels were also measured using western blot. As shown in Fig. 1b, Chm-1 protein was specifically expressed in chondrocytes at a level higher than that of MSCs (*P* < 0.01). Immunofluorescence tests further demonstrated that the expression of Chm-1 differed in chondrocytes and MSCs (Fig. 1c). The result demonstrated that Chm-1 was specifically expressed in chondrocytes.

### Transfection of MSCs with *Chm-1* gene

As shown in Fig. 1a, the *Chm-1*, *Col II*, and *AGG* genes were detected by RT-PCR in the T-MSCs, whereas the expression of *Chm-1* gene was very low in the untransfected MSCs. To further confirm chondrogenic phenotype on a protein level, Chm-1, Col II, and AGG protein levels were also measured using western blot. As shown in Fig. 1b, Chm-1 was specifically expressed in T-MSCs at a level higher than that of untransfected MSCs (*P* < 0.01). Meanwhile, Col II and AGG were significantly expressed in T-MSCs at levels higher than those of MSCs (*P* < 0.01). An immunofluorescence observation (Fig. 1c) indicated that T-MSCs expressed Chm-1 and Col II, whereas no staining could be observed in untransfected MSCs. These findings indicated that adenovirus-mediated transfection successfully generated Chm-1 gene-modified MSCs.

### Construction of cell–scaffold complex

**Fig. 2:**
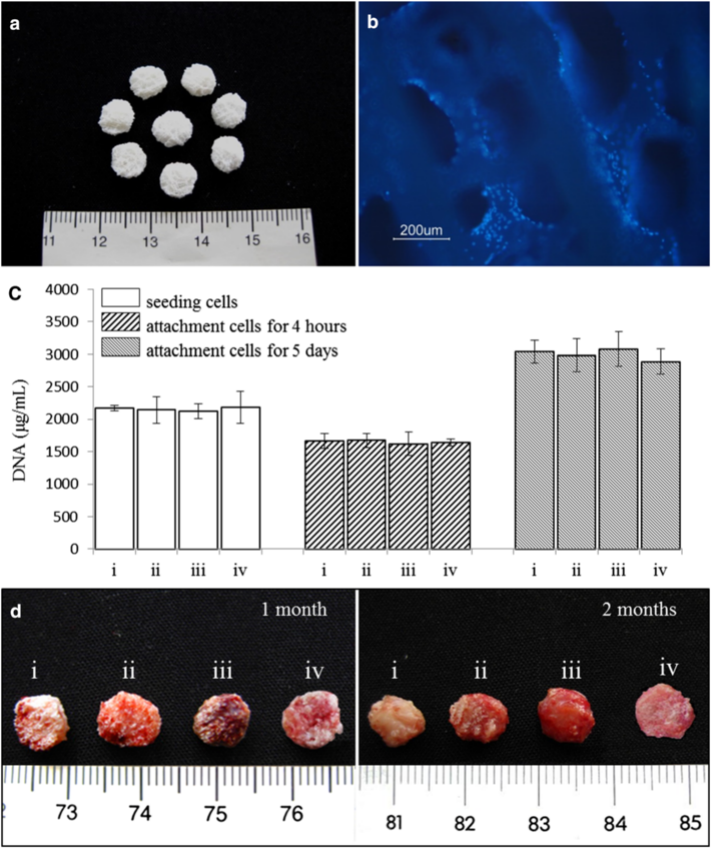
**a** Natural coral scaffolds (8 mm in diameter and 2 mm in height). **b** T-MSCs and coral scaffold complex. Fluorescence microscope examination showed the attachment of T-MSCs on the coral scaffold. Nuclei were visualized by Hoechst 33342 staining. **c** Cell-seeding efficacy and proliferation on coral scaffolds (*n* = 4). Each scaffold was seeded with 2 × 10^6^ cells which were *i* chondrocytes, *ii* 1:1 mixture of MSCs and chondrocytes, *iii* MSCs, and *iv* T-MSCs. The initial 2 × 10^6^ cells before seeding from each group acted as control. There were no significant differences among groups (*P* > 0.05). **d** Representative macroscopic pictures of the cell–scaffold composites (*i* chondrocyte–coral composites, *ii* chondrocytes and MSCs coseeded into natural coral scaffolds in ratio of 1:1, *iii* MSC–coral composites, and *iv* T-MSC–coral composites) removed from animals after 1 month and 2 months (Color figure online)

The coral scaffold is shown in Fig. 2a. Approximately 2 × 10^6^ cells were seeded on each coral scaffold to form a cell–scaffold complex (Each scaffold loaded with  i) chondrocytes, ii) 1:1 mixture of MSCs and chondrocytes, iii) MSCs, and iv) T-MSCs, respectively.). According to Fig. 2c, cell-seeding efficiency was around 75–78 %, and cells proliferated 1.32–1.45 times on coral scaffolds 5 days after seeding in each group. There were no significant differences among groups. Cell nuclei were stained with Hoechst 33342 and visualized by fluorescence microscope. The fluorescent micrographs (Fig. 2b) indicated that the internal structure of the coral scaffold was porous, and that cells were evenly distributed throughout the coral scaffolds. The results indicated that the scaffold was biocompatible and able to support the initial attachment and subsequent proliferation of MSCs in vitro.

### In-vivo evaluation of the tissue-engineered cartilage

Figure 2d shows the gross appearance of specimens from different groups 1 and 2 months after implantation. Specimens from the chondrocyte-seeding (Fig. 2d,*i*) and chondrocyte–MSC-coseeding (Fig. 2d,*ii*) groups appeared light red. Specimens from the MSC-seeding group (Fig. 2d,*iii*) were dark red, and a thin layer of soft tissue and blood vessels could be observed clearly on the surfaces of the specimens. In contrast, specimens from the Chm-1-transfected MSCs (Fig. 2d,*iv*) could be separated easily from the adherent fibrous capsule and were light red.

**Fig. 3:**
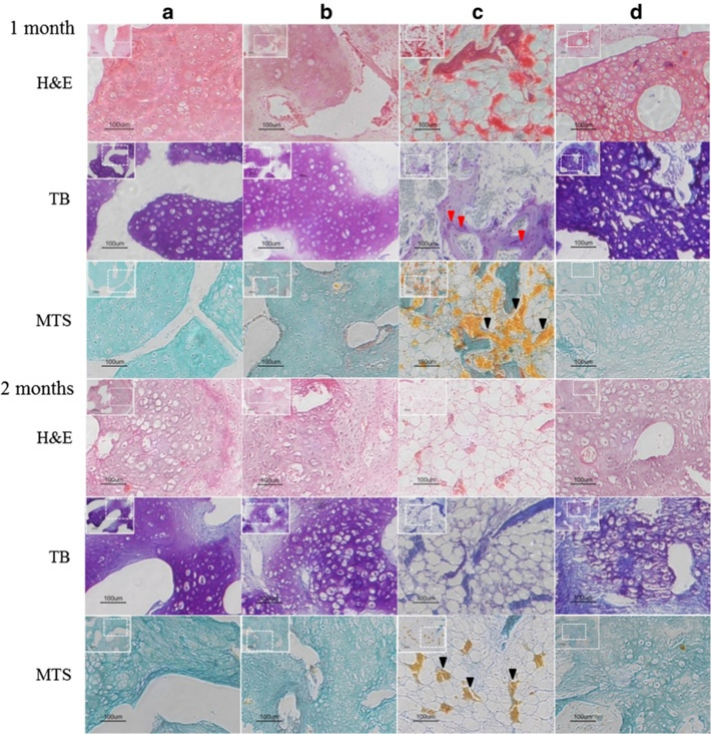
Histologic analyses in chondrogenically differentiated cells and the vascularization in newly formed tissue in vivo. The specimens were transplanted into nude mice. Appearance of specimens 1 month and 2 months post transplantation for **a** chondrocyte–coral composites, **b** chondrocytes and MSCs coseeded into natural coral scaffolds in a ratio of 1:1, **c** MSC–coral composites, and **d** T-MSC–coral composites. The specimens were processed for histologic staining with H & E, TB, and MTS. *Red arrows* indicate endochondral ossification. *Black arrows* indicate vascular structures. *H&E* hematoxylin and eosin, *MTS* Masson’s trichrome staining, *TB* toluidine blue (Color figure online)

H & E staining, TB staining, and MTS were performed to examine the tissue formation and vessel density in the scaffolds. H & E staining, TB staining, and MTS observation did not reveal obvious vascularization and bone formation in the chondrocyte-seeding (Fig. 3a) and chondrocyte–MSC-coseeding (Fig. 3b) groups. However, TB staining showed mature bone formation via endochondral ossification in scaffolds seeded only with MSCs (Fig. 3c, arrow), and MTS results (Fig. 3c) revealed active vascularization (arrow) in these specimens. By contrast, a large amount of mature cartilage formed in the pores of the coral scaffold, and bone formation and vascularization were not evident in the T-MSC specimens (Fig. 3d).

**Fig. 4:**
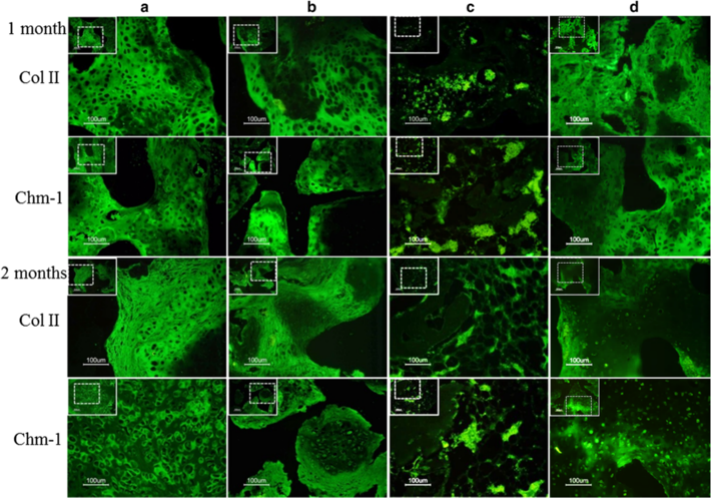
Analysis of chondrogenic protein expression for neocartilage formation in the specimens. The specimens were transplanted into nude mice. Specimens 1 and 2 months post transplantation: **a** chondrocyte–coral composites; **b** chondrocytes and MSCs coseeded into natural coral scaffolds in a ratio of 1:1; **c** MSC–coral composites; and **d** T-MSC–coral composites. Specimens were processed to analyze the distribution of Col II (*green*) and Chm-1 (*green*) in cells by immunofluorescence (Color figure online)

Expression of the chondrogenic-specific proteins Col II and Chm-1 was evaluated by immunohistology. As showed in Fig. 4, Col II (green fluorescence) and Chm-1 (green fluorescence) expression were evident in the chondrocyte-seeding (Fig. 4a) and chondrocyte–MSC-coseeding (Fig. 4b) groups but rarely in scaffolds seeded only with MSCs (Fig. 4c). The T-MSCs expressed abundant Col II (green fluorescence) and Chm-1 (green fluorescence), which was evenly distributed throughout the cells (Fig. 4d).

**Fig. 5:**
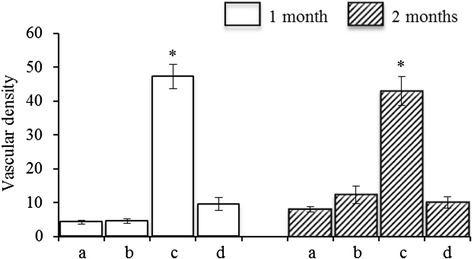
Number of vessels in the specimens 1 month and 2 months post transplantation were statistically analyzed: **a** chondrocyte–coral composites; **b** chondrocytes and MSCs coseeded into natural coral scaffolds in a ratio of 1:1; **c** MSC–coral composites; and **d** T-MSC–coral composites. Vascular numbers of each specimen were counted per time point as the mean ± SD. Each bar represented four specimens (*P < 0.05)

MTS-stained vessels were observed in the specimens after implantation for 1 and 2 months (Fig. 5). The blood vessel density of both groups continuously increased 1–2 months after implantation. Compared with the specimens seeded with MSCs (1 month 47.25 ± 3.59; 2 months 43.00 ± 4.24), the mean blood vessel densities in the chondrocyte–MSC-coseeding groups (1 month 4.50 ± 0.67; 2 months 12.25 ± 2.58) were significantly reduced at each time point (*P* < 0.05). The mean blood vessel density in the chondrocytes:MSCs = 1:1 group did not significantly differ from that in the chondrocyte-seeding group (1 month 4.25 ± 0.53; 2 months 8.00 ± 0.82; *P* > 0.05). MTS indicated that the mean blood vessel density in the T-MSC-seeding group (1 month 9.58 ± 1.85; 2 months 10.08 ± 1.64) was significantly lower than that in the MSC-seeding group (1 month 47.25 ± 3.59; 2 months 43.00 ± 4.24) at each time point (*P* < 0.05).

## Discussion

MSCs could undergo chondrogenic differentiation in the presence of the appropriate growth factors and has been considered an attractive cell source for cartilage tissue engineering [8]. However, tissue-engineered cartilage constructed by chondrogenic induction of MSCs exhibits a hypertrophic phenotype and extensive calcification of the extracellular matrix after implantation [12, 13]. Mueller and Tuan [33] reported that the combination of TGF-β withdrawal, dexamethasone reduction, and thyroid hormone addition could induce hypertrophy of chondrogenic induced MSCs, accompanied by increased alkaline phosphatase activity, matrix mineralization, and changes in hypertrophy markers. They concluded that chondrogenically induced MSCs were functionally similar to growth plate chondrocytes, which underwent a differentiation program analogous to endochondral ossification. Vascular invasion is a histological marker of endochondral ossification, and previous studies show that the degree of vascularization significantly differs between cartilage engineered with chondrocytes and cartilage engineered with MSCs [34], which indicate that the regulation of vascular formation may differ greatly between chondrocytes and MSCs. These drawbacks may significantly influence the treatment of cartilage defects with MSCs. Chm-1 is specifically expressed in the avascular area of some mesenchymal tissues, including certain ocular tissues, cardiac valve, and cartilage [23, 35–38]. Chm-1 has been demonstrated to inhibit angiogenesis, and many studies investigate the effects of Chm-1 and the mechanisms by which it inhibits angiogenesis and disrupts the vasculature [39–41].

Microfracturing of the subchondral bone plate is a frequently employed approach in the clinic. The approach could guide MSCs to migrate into the defect to improve cartilage lesion repair [42]. Blanke et al. [25] reported that cartilage healing from microfracturing lacked the expression of Chm-1, and was associated with excessive matrix calcification and vascular ingrowth. And that additional transplantation of chondrocytes could significantly prevent matrix calcification and vascular ingrowth. In agreement with Blanke et al.’s study, we found that MSCs expressed cartilage-specific genes including *Col II* and *AGG* (Fig. 1), however, unlike chondrocytes which expressed high levels of *Chm-1,* the expression of *Chm-1* was very low in MSCs (Fig. 1). This finding further demonstrated that native MSCs are not optimized cells for cartilage regeneration.

We then transfected MSCs with Ad5-Chm-1. Interestingly, transfected MSCs not only expressed a high level of Chm-1 but the expression of cartilage-specific genes was also significantly upregulated (Fig. 1). Previous studies have indicated that coculturing MSCs with chondrocytes leads to increased chondrogenic gene expression and ECM deposition in MSCs, and these phenotypic changes are considered to be the result of growth factors secreted by chondrocytes [43, 44]. According to the result of our study, Chm-1 is also a signaling molecule that regulates chondrogenic phenotype of MSCs.

Finally, we fabricated cartilage grafts with Chm-1-transfected MSCs. In this experiment, we used porous coral as a cell-seeding scaffold, which facilitates the vascularization and ossification of engineered tissue [45, 46]. The result showed that mature cartilage formed in the pores of the scaffold, and bone formation was not observed in the specimens 2 months after implantation (Figs. 2, 3, 4). The newly formed tissue also exhibited strong immunohistochemical staining for Chm-1. However, a large amount of bone and marrow tissue formed in the MSC-seeding group 2 months after implantation (Figs. 2, 3, 4). Importantly, the number of blood vessels in the engineered graft was similar to that in the chondrocyte-seeding group (*P* > 0.05) and significantly lower than that in the MSC-seeding group (*P* < 0.05) (Fig. 5). Klinger et al. [47] transfected osteochondral progenitor cells with Chm-1 and subsequently transplanted cells into cartilage lesions of a joint and found that transfected osteochondral progenitor cells maintained chondrogenic phenotype and formed hyaline cartilage. The avascular and hypoxia environment of the joint made it difficult for them to obtain quantified data for the antivascular effect of Chm-1. Compared with Klinger et al., we ectopically implanted Chm-1-transfected MSCs with coral scaffold. Our results indicated that Chm-1 is critical in inhibiting vascularization and maintaining the chondrogenic phenotype of tissue-engineered cartilage from MSCs, even in a microenvironment suitable for tissue vascularization and ossification.

Several genes have been considered as targets to facilitate cartilage formation by autologous cells, including TGF-β1, BMPs, IGF, and FGF-2 [43, 44]. The results of the current experiment indicated that *Chm-1* could not only upregulate chondrogenic phenotype of MSCs but could also prevent vascularization. Consequently, this gene efficiently maintained the chondrogenic phenotype of engineered cartilage. We conclude that Chm-1 gene-modified MSCs may hold great potential in tissue-engineering applications for cartilage regeneration.

## Conclusions

In summary, we transfected rabbit MSCs with Ad5-Chm-1 and seeded these cells into a natural coral tissue-engineering scaffold to investigate the effect of exogenous Chm-1 expression in MSCs. We report that Chm-1 inhibited vascularization and maintained chondrocyte phenotype in vivo in Ad5-Chm-1-transfected MSCs. These results demonstrated that Chm-1-modified MSCs may be an optimized cell source for cartilage tissue engineering.

## Abbreviations

Ad5-Chm-1: adenovirus-containing Chm-1 gene
bp: base pairs
Chm-1: Chondromodulin-1
FMMU: Four Military Medical University
GFP: green fluorescent protein
H & E: hematoxylin and eosin
MOI: multiplicity of infection
MSC: mesenchymal stem cell
MTS: Masson’s trichrome staining
RT-PCR: reverse transcription-PCR
TB: toluidine blue
TGF-β: transforming growth factor beta
T-MSC: Ad5-Chm-1-transfected mesenchymal stem cell
